# Mucosal associated invariant T cells: Powerhouses of the lung

**DOI:** 10.1016/j.imlet.2024.106910

**Published:** 2024-10

**Authors:** J.C. López-Rodríguez, P. Barral

**Affiliations:** a Centre for Inflammation Biology and Cancer Immunology, The Peter Gorer Department of Immunobiology, King's College London, London, UK; b The Francis Crick Institute, London, UK

**Keywords:** MAIT cells, Lung immunity, MR1, Infection, Inflammation

## Abstract

The lungs face constant environmental challenges from harmless molecules, airborne pathogens and harmful agents that can damage the tissue. The lungs’ immune system includes numerous tissue-resident lymphocytes that contribute to maintain tissue homeostasis and to the early initiation of immune responses. Amongst tissue-resident lymphocytes, Mucosal Associated Invariant T (MAIT) cells are present in human and murine lungs and emerging evidence supports their contribution to immune responses during infections, chronic inflammatory disorders and cancer. This review explores the mechanisms underpinning MAIT cell functions in the airways, their impact on lung immunity and the potential for targeting pulmonary MAIT cells in a therapeutic context.

## Highlights

• Pulmonary MAIT cell activation can be mediated by TCR-dependent antigen recognition and/or cytokines.
• Activated MAIT cells accumulate in the lung and provide protection against bacterial and viral infections.
• Pulmonary MAIT cells may be involved in chronic pulmonary disorders and tumour immunity.
• The pulmonary MAIT cell population might be boosted for clinical benefit in vaccination or therapeutic strategies.

## 1 Introduction

The lungs are continually exposed to environmental challenges including non-harmful molecules, airborne pathogens, and harmful agents such as chemicals that can damage the lung tissue. The lung's immune system is essential for defense against respiratory pathogens while also tolerating non-harmful aeroantigens. Accordingly, the lungs host a vast population of immune cells, including large numbers of tissue-resident lymphocytes which provide a first line of defense against external insults and contribute to maintaining the balance between immunity and tolerance. Whilst much research in lung immunity has focused on the function of conventional MHC-restricted T cells, recent studies suggest important roles for Mucosal Associated Invariant T (MAIT) cells as key players in the airways [[1], [2], [3], [4]]. MAIT cells express a semi-invariant T cell receptor (TCR) which recognises antigens derived from the riboflavin synthesis pathway presented by the MHC-class 1b related molecule 1 (MR1). The strong evolutionary conservation of the MR1 antigen presentation system and the MAIT cell transcriptional program suggest important and non-redundant roles for MAIT cells in the regulation of immunity [[5], [6], [7]]. However, despite the relative abundance of MAIT cells in the human and murine lung, identifying their unique functions in this tissue has been challenging. The causal relationship between pulmonary MAIT cell functions and immunopathology only recently started to come to light with an increasing number of manuscripts presenting data on murine models of infectious, inflammatory lung diseases and cancer. The paucity of these studies is likely related to the low numbers of MAIT cells found in most laboratory mouse strains, in addition to the complementary/compensatory functions of various innate immune cells which complicate the identification of specific MAIT cell functions. Despite these challenges, increasing evidence support non-redundant roles for MAIT cells in immune responses during pulmonary bacterial and viral infections, in chronic pulmonary disorders and in tumour immunity. In this review we provide an update on the current understanding of the mechanisms underlying MAIT cell functions in the lungs and discuss how these cells contribute to the regulation of immunity and shape disease severity in the airways. Moreover, we will examine the available evidence supporting the targeting of pulmonary MAIT cells for the design of new vaccination and therapeutic strategies.

## 2 Pulmonary MAIT cells: properties, distribution and activation

Originally identified as CD8^-^CD4^-^ T cells in the peripheral blood of healthy individuals [8], this abundant subset of unconventional T cells were named as ‘MAIT cells’ in 2003, when *Treiner et al*. described a novel subset of resident T cells in the intestinal lamina propria [9]. Unlike conventional T cells, MAIT cells express a semi-invariant TCRα chain (Vα7.2-Jα33/Jα12/Jα20 in humans, Vα19-Jα33 in mice) which combines with a limited set of β-chains (Vβ6/Vβ8 in mice, Vβ2/Vβ13 in humans) [[10], [11], [12], [13]]. The MAIT-TCR is restricted by MR1, a highly conserved molecule which shares the common MHC-I folding structure and high degree of homology between human and mouse [9,[14], [15], [16]]. Thymic development of MAIT cells strictly depends on MR1 expression by cortical thymocytes and it is shaped by microbiota-derived signals as well as expression of the transcription factor promyelocytic leukaemia zinc finger (PLZF) [[17], [18], [19], [20], [21]]. It is important to note that there are T cells that recognize MR1 but do not fit with the canonical definition of MAIT cells as described above [22] and won't be covered in this review. These atypical/non-canonical MR1 restricted T cells are much less abundant than MAIT cells, express a different TCR, and recognize different ligands [22].

### 2.1 Phenotype of pulmonary MAIT cells

MAIT cells are found in the circulation and in virtually all tissues, but they are particularly enriched in mucosal organs, especially in the lungs. In humans, MAIT cells represent ∼6–10 % of T cells in the blood, while in the lungs their frequency increases up to 15 %. MAIT cell numbers are low in mice, yet cells exhibit a similar tissue distribution to their human counterparts accounting for ∼0.1 % of total T cells in blood and 3–5 % in the lungs [[23], [24], [25], [26]]. The phenotype of MAIT cells is tissue- and species-specific, with cells being predominantly CD4^-^CD8^-^ in mice, whereas CD8^+^ MAIT cells are prevalent in humans, although CD4^-^CD8^-^ MAIT cells are also found in the human lung [[25], [26], [27]]. Both pulmonary murine and human MAIT cells show a marked tissue residency phenotype, characterised by the expression of a wide set of homing receptors and a “tissue-resident” transcriptional program [[28], [29], [30]]. In keeping with this, mouse parabiotic experiments demonstrate that lung MAIT cells are a predominantly tissue-resident population [28], although the mechanisms involved in lung-retention remain undefined.

In peripheral tissues of mice, we can find several functional subsets of MAIT cells that are classified on the basis of their transcription factor expression and cytokine secretion: MAIT1 are T-bet^+^ and primarily secrete IFN-γ upon activation, while MAIT17 cells express RORγt^+^ and produce IL-17 [17,25]. These subsets are found at different proportions in various tissues in homeostatic conditions with MAIT17 representing up to 90 % of MAIT cells in the murine lung [28]. However, the functional MAIT1/MAIT17 bifurcation is absent in humans, and human pulmonary MAIT cells display a *hybrid* phenotype with a mixed gene expression pattern [29,31]. Interestingly, this hybrid phenotype can be also found in activated murine pulmonary MAIT cells as it is the case during infection with *Salmonella typhimurium* or *Legionella longbeachae,* in which cells co-express RORγt and T-bet [[32], [33], [34]]. Conversely, a transcriptionally distinct MAIT17-like population has been detected in the bronchoalveolar lavage of children hospitalised with community acquired pneumonia, although these cells also express *TBET* [35]. Thus, it is possible that MAIT cells exhibit a degree of functional plasticity driven by environmental and/or inflammatory signals in the tissues. Nevertheless, the nature of such signals and the origin (resident, circulating) and functional features of the MAIT cell populations arising in response to specific challenges is a matter of investigation.

### 2.2 Anatomical location of pulmonary MAIT cells

**Fig. 1:**
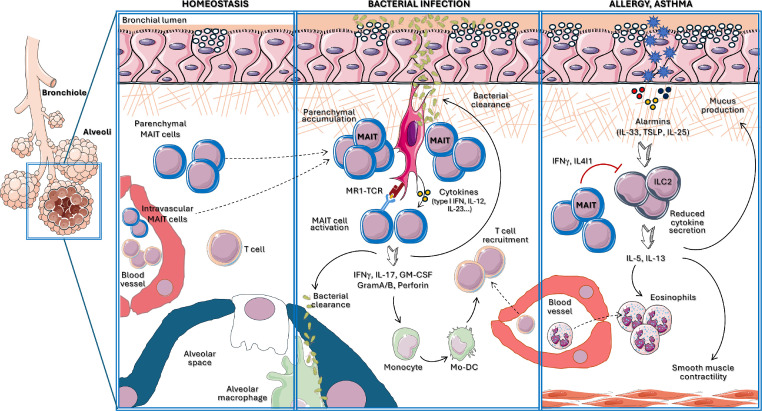
**MAIT cells play protective roles in pulmonary infection and inflammation.** In homeostasis (left), MAIT cells are primarily located in the lung parenchyma (mainly MAIT17 cells), although MAIT1 cells are also found in the vasculature. During bacterial infection (middle) pulmonary MAIT cells are activated via MR1-antigen presentation and/or cytokines (e.g. IL-23, IL-12, type I IFN), and cells expand and accumulate in the lung parenchyma. Upon activation, MAIT cells secrete a wide range of cytokines (e.g. IFN-γ, IL-17, GM-CSF) and cytolytic agents (granzymes, perforin) by which they can modulate the recruitment and activation of other immune cells in the airways and drive pathogen clearance. MAIT cells play a protective role during chronic airway inflammation such as allergy and asthma (right). In this context, MAIT cells secreted mediators such as IL4I1 or IFN-γ, repress ILC2-mediated production of IL-5 and IL-13, eosinophil recruitment and subsequent airway inflammation.

Within the lung tissue, immune cells are found across diverse anatomical spaces including the alveoli (air space), the vasculature and the airway parenchyma, the latter being a few microns-width gap which separates the alveoli and the vasculature [[36], [37], [38]]. The alveoli house a small number of immune cells, comprised almost entirely by alveolar macrophages, while T cells are mainly found in the parenchyma and within the vasculature. The specific location and migration of immune cells within the lung tissue will shape the promptness and effectivity of their functions, a process that is under the control of many local factors such as cytokines and chemokines. While the distribution and localisation of MAIT cells within the airways remain poorly explored, recent studies suggest that in homeostasis murine and human cells are located primarily in the lung parenchyma [32,39] (Fig. 1). Moreover, MAIT cell anatomical location is defined by their functional phenotype [28], and while MAIT17 cells present a parenchymal location in homeostasis, MAIT1 cells are primarily found within the vasculature [28,32]. It is important to mention that the (micro)anatomical location of pulmonary MAIT cells changes under pathogenic conditions. For instance, in response to *Klebsiella pneumoniae* infection murine MAIT cells accumulate in peri‑bronchial spaces [39]. Moreover, MAIT cells have been detected in lung lesions from patients infected with *Mycobacterium tuberculosis* [40], and in close proximity to *Legionella longbeachae* in the lung parenchyma after infection of human lung tissue *ex vivo* [32].

### 2.3 Activation of pulmonary MAIT cells

Due to their peripheral location, MAIT cells act at the frontline in response to a wide variety of pathogens and environmental challenges and are activated early during immune responses. Indeed, it seems likely that a main reason for MAIT cell evolutionary conservation and their abundance in lung tissues, is their key role in early immunity. MAIT cells are activated by TCR-dependent recognition of metabolites derived from the riboflavin synthesis pathway which are produced by several strains of bacteria and fungi [41]. In addition to MR1-TCR–dependent activation, MAIT cells can also be activated in response to cytokines through MR1-independent mechanisms [42,43]. This is the case during viral infections as well as some bacterial infections in which cytokines such as IL-18, IL-12, and type I IFNs are sufficient to stimulate MAIT cells [39,44]. Moreover, cytokines can synergize with TCR signals to ultimately increase MAIT cell effector functions [33,45]. Despite the increasingly well-characterised functions of MAIT cells, the mechanisms driving their activation and effector functions in the tissues remain poorly defined and seem to be insult-dependent and likely modulated by local tissue-specific signals as discussed below.

## 3 Identifying the functions of pulmonary MAIT cells: challenges and limitations

Despite the relative abundance of MAIT cells in the lung, a causal relationship between pulmonary MAIT cells and immunopathology has only recently started to emerge. While data on respiratory bacterial infections is increasing [46], the protective or detrimental roles for MAIT cells in chronic pulmonary inflammatory diseases or cancer remain an open question. The paucity of these studies is likely related to a variety of factors including the low numbers of MAIT cells found in most laboratory mouse strains [23,47], the interspecies variation in terms of numbers, phenotype and functional capacity found for murine vs. human MAIT cells [47], and the complementary/compensatory functions for various innate immune cell populations [17,48].

MAIT cells are scarce in most laboratory mouse strains breed under specific pathogen free conditions [23,47] and virtually absent in germ-free mice [9,17,20], while they are abundant in the human lung [26]. Thus, it is likely that MAIT cells play critical and non-redundant roles in human pulmonary diseases which may not be fully recapitulated (and possibly overlooked) in inbred murine models. Moreover, it has been suggested that the various unconventional T cell populations (including MAIT, NKT and γδ T cells) compete for a shared environmental niche [17,48] leading to possible compensatory mechanisms which will further mask the unique functions of the specific subsets *in vivo*. In keeping with this, a recent case study reported an individual with a point mutation in MR1 and a lack of circulating MAIT cells who has a history of difficult to treat viral and bacterial infections, supporting an integral role for MAIT cell in host protection [49]. The patient had an expanded γδ T cell population, indicating a compensatory interplay between unconventional T cell subsets also occurring in humans.

The functions of pulmonary MAIT cells have been studied using a variety of mouse strains and approaches to either delete or increase MAIT cell numbers [47], although several of these models present further alterations which may confound the interpretation of some experimental data. Amongst transgenic mice, MR1-KO [9] is the more popular strain due to its complete lack of MAIT cells, although MR1-restricted non-MAIT T cells are also depleted in these animals. Traj33-deficient mice have been also generated and show a strong reduction in the number of MAIT cells, yet some residual antigen-reactive cells persist in these mice [50]. On the other hand, MAIT cells are increased in Vα19 TCR transgenic mice, but these cells are phenotypically and functionally different to MAIT cells identified in WT mice [25]. Also, the CAST/EiJ strain and the congenic B6-MAIT^CAST^ strain show an increase in the numbers of pulmonary MAIT cells in comparison with WT C57BL/6 mice [23,51]. Notably, B6-MAIT^CAST^ in an MR1-KO background (B6-MAIT^CAST^MR1^-/-^) show an increase in the absolute numbers of pulmonary NK cells which could compensate for the lack of MAIT cell functions in some settings [51]. Finally, several approaches have been used to expand the pulmonary MAIT cell population in mice. These include the intranasal administration of the MAIT cell antigen 5-OP-RU (5-(2-oxopropylideneamino)-6-d-ribitylaminouracil) together with cytokines or TLR ligands [33,34]. Also, several bacterial pathogens when administered intranasally induce an accumulation of MAIT cells in the murine lung. One laboratory model widely employed in the field is the intranasal administration of the enteropathogen *Salmonella* enterica serovar *Typhimurium*, which induces the accumulation and activation of pulmonary MAIT cells [34]. It is important to note that these expanded antigen experienced MAIT cells show a different phenotype and transcriptome when compared to steady-state pulmonary cells [33,52].

## 4 MAIT cells as sentinels during respiratory bacterial infections

Initial reports using human MAIT cells isolated from peripheral blood or murine splenic MAIT cells demonstrated that these cells can be efficiently activated by airborne bacteria suggesting a role for MAIT cells in respiratory bacterial infections [40,53]. Subsequently, several models of murine bacterial pneumonia highlight key and non-redundant protective roles for MAIT cells underpinned by diverse (MR1-dependent and independent) mechanisms of activation as summarised below (Fig. 1).

Several riboflavin-producing bacteria have been shown to activate MAIT cells in an MR1-dependent manner, ultimately leading to protective roles for MAIT cells in bacterial pneumonia. Some of the best characterised examples include the live vaccine strain (LVS) of *Francisella tularensis* [54,55] as well as *Legionella longbeachae* [32,33]*.* MAIT cells accumulate in the murine lung after intranasal infection with these bacteria, and optimal MAIT cell activation and expansion is MR1-dependent and supported by cytokines such as IL-12 in the case of *Francisella* [54] and IL-23 for *Legionella* [33]. MR1-KO mice intranasally infected with these bacteria show a higher bacterial burden in comparison to WT counterparts, demonstrating a non-redundant role for MAIT cells in protection against *Francisella* and *Legionella*. Mechanistically, protection from *Legionella* depends on IFN-γ production by MAIT cells [32], while during *F. tularensis* LVS infection MAIT cells promote early pulmonary GM-CSF production, which drives the differentiation of inflammatory monocytes into dendritic cells, recruitment of T cells to the lungs, and control of pulmonary bacterial growth [55]. In keeping with this, MAIT cells also contribute to protection during systemic infection of mice with *F. tularensis* LVS, with bacterial infection inducing a significant expansion of MAIT1 cells (T-bet^+^RORγt^-^) in peripheral organs including the lungs [56]. Another example of MR1-driven MAIT cell activation in the lungs occurs in response to murine pulmonary infection with *Salmonella* enterica serovar *Typhimurium* [34]. *S. Typhimurium*, induces the accumulation and activation of pulmonary MAIT cells in a process that is dependent on MR1 and supported by signalling through the costimulatory molecule ICOS and IL-23 [33,34]. However, bacterial lung clearance in this model is independent of the presence of MAIT cells, suggesting their redundancy as protective cells in this murine system [34]. Interestingly, following pulmonary infection with *Salmonella*, mouse MAIT cells expand in the lungs and generate two populations with distinct effector functions, metabolism and transcriptional programs characterised by the different expression of CD127 and Klrg1 [52]. These antigen experienced MAIT cells confer protection to pulmonary infection: CD127^+^ MAIT cells protect mice against *Streptococcus pneumoniae* infection in an MR1-dependent manner, while both CD127^+^ and Klrg1^+^ MAIT cells improve survival during influenza infection [52]. In keeping with the murine data, human studies also support a protective role for MAIT cells during *S. pneumonia* infection, as in a human challenge increased blood MAIT baseline responses correlate with protection against mucosal colonisation [57]. Interestingly, human and murine MAIT cell response towards *S. pneumoniae* clinical isolates is different according to the bacterial expression of riboflavin pathway genes [58], suggesting alterations in this pathway as a potential immune-evasion strategy.

In addition to the MR1-dependent activation of pulmonary MAIT cells described above, recent reports propose that cytokines are sufficient to drive MAIT cell activation during bacterial pneumonia. For example, our group has recently identified a role for MAIT cells during pulmonary infection with *Klebsiella pneumoniae* [39]. Even though *Klebsiella* encodes genes involved in riboflavin biosynthesis, we found that this bacterium drives activation and a Th1-like transcriptional program in both human circulating and murine pulmonary MAIT cells through a mechanism regulated by type I IFN, independently of MR1-TCR signals. In support of this, transfer of MAIT cells to immune-deficient or MR1-deficient mice protect from *Klebsiella pneumoniae* infection, with protection being dependent on direct type I IFN signalling on MAIT cells [39]. In keeping with these data, another report demonstrated that MR1-KO mice infected intraperitoneally with *Klebsiella pneumoniae* showed decreased survival and failed to control bacterial burden at early time-points after infection [59], although the activation and effector functions of MAIT cells in this model or the mechanisms mediating protection haven't been addressed. Another example of MAIT cell driven protection during bacterial pneumonia includes the aerosol-mediated infection with *Mycobacterium bovis BCG* in which MR1-KO mice showed higher lung bacterial burden than WT counterparts at early time-points after infection [60]. While the mechanisms underpinning MAIT cell activation and bacterial control *in vivo* remain unclear, *in vitro* experiments suggest that MAIT cell effector functions in response to *M. bovis BCG* are MR1-independent and primarily driven by IL-12 and IL-18 [60,61].

## 5 MAIT cells in respiratory viral infections

To date, less is known about the role of MAIT cells in responses to respiratory viral infections in comparison to bacteria. While viruses do not produce MR1-ligands, viral infections efficiently induce MAIT cell activation mediated by a variety of cytokines secreted by host cells such as IL-12, IL-15, IL-18 or type-I IFNs [44]. The best characterised model of MAIT cell responses to respiratory viruses is that of influenza which leads to activation and cytokine secretion by both human and murine MAIT cells. Indeed, MAIT cells from human peripheral blood are activated by influenza A virus in a process mediated by IL-18 and resulting in robust IFN-γ and Granzyme B secretion by MAIT cells [44,62]. Moreover, reduced numbers of circulating MAIT cells correlate with disease severity in patients infected with influenza virus [44,62,63], suggesting a protective role for MAIT cells during influenza infection. This MAIT cell-mediated protection was further confirmed in mice infected with the influenza virus strain A/Puerto Rico/8/34/1934 (PR8, H1N1), which causes severe pneumonia. MR1-KO mice intranasally infected with PR8 show impaired survival in comparison to WT counterparts, while survival improves by adoptive transfer of pulmonary MAIT cells to immunocompetent and immunodeficient mice prior to infection [63]. In this model, MAIT cells accumulate in the lung and show signs of activation at early time-points after infection. Both of these processes occurred in an MR1-independent manner and are primarily mediated by cytokines including IL-18, which controls MAIT cell accumulation in the lungs, whilst a variety of cytokines (IL-18, IL-12, IL-15, type I IFNs) influence MAIT cell activation. Mechanistically, the antiviral effects of MAIT cells were mediated -at least in part- by IFN-γ [63]. In keeping with these results -and as mentioned above- antigen experienced MAIT cells (generated after exposure to *S. Typhimurium*) also conferred a survival benefit when transferred to recipient mice prior to infection with PR8 [52].

Another extensively studied MAIT cell response to respiratory virus is SARS-CoV-2 and its associated coronavirus disease 2019 (COVID-19). As observed with other lung diseases, a reduced frequency of circulating MAIT cells was observed in COVID-19 patients while cells accumulated in the lung and show a strong activated and cytotoxic phenotype [[64], [65], [66], [67]]. In this context, MAIT cell activation and phenotype are associated with COVID-19 severity, clinical parameters and disease outcome. Moreover, in a SARS-CoV-2 human challenge study, blood MAIT cells were found to be activated in sustained infections, but also in abortive and transient infections [67]. Mechanistically, MAIT cell activation and effector functions have been proposed to be regulated by an IFN-α–IL-18 imbalance associated with disease severity. Thus, while MAIT cell activation at early stages of infection may be driven by type I IFN, in severe disease there is switch towards IL-18 which will further drive MAIT cell functions [65]. After infection, MAIT cell numbers and phenotype recover in the circulation within weeks of resolution of COVID-19 symptoms, although longer follow up (9 months) shows that recovery is not sustained and MAIT cell numbers decrease again with remaining cells being functionally impaired and showing an exhausted phenotype in a subset of patients [64,68]. Thus, while a large amount of data supports the participation of MAIT cells in the immunopathology of COVID-19, whether these cells play a protective or pathogenic role at various stages of the disease remains to be elucidated.

## 6 MAIT cells in non-infectious lung disorders

In addition to MAIT cells’ roles in respiratory infections, emerging evidence suggests that these cells may be also involved in non-communicable pulmonary diseases such as cancer and chronic inflammatory diseases. Among others, those include asthma, chronic obstructive pulmonary disease (COPD) and cystic fibrosis (CF), which in general are symptomatically characterised by a profound lung tissue inflammation, mucus hypersecretion and airway hyperresponsiveness (AHR) and remodelling, which can eventually lead to respiratory failure. Overall, while the frequency and phenotype of pulmonary (and/or circulating) MAIT cells are altered in these disorders, whether these cells contribute to disease initiation or progression, or whether they are activated as a result of the inflammatory environment remains an open question.

Asthma is defined as a chronic inflammation of the airways mainly linked to a Th2 exacerbated responses in the lungs. The most common clinical form of asthma is called allergic asthma and is driven by IgE-mediated allergic sensitisation against inhaled or ingested allergens from sources such as house dust mites, plant pollen or peanuts. Initial studies suggest a protective role for MAIT cells in the immunopathology of asthma as a higher frequency of MAIT cells in the peripheral blood of 1 year-old children has been associated with a decreased risk of asthma by age seven years [69]. Moreover, the frequency of MAIT cells in peripheral blood, sputum and endobronchial biopsies is severely reduced in asthmatic patients [70], although it remains unclear whether such changes are related to corticosteroid therapy [70]. A causal relationship between MAIT cells and allergic asthma has been established in mice [71,72]. Sensitisation of mice with a variety of common allergenic sources (e.g. house dust mite, *Aspergillus, Alternaria*) results in reduced numbers of pulmonary MAIT cells, as observed in humans [71]. Functionally, MAIT cells repress allergic airway inflammation and AHR, as MR1-KO mice show increased eosinophil lung infiltration and AHR in models of acute *Alternaria* inhalation [71]. Complementary results were obtained after *Alternaria* challenge of Vα19 TCR transgenic mice which presented decreased lung eosinophilia and reduced airway inflammation in comparison with WT [72]. Mechanistically this process is mediated by the MAIT cell-dependent repression of group 2 innate lymphoid cells (ILC2), which show increased expression of IL-5 and IL-13 in MR1-deficient animals [71,72] (Fig. 1). Thus, it is possible that the reduced numbers of MAIT cells found in asthmatic patients may contribute to an exacerbated inflammatory response to allergens. In addition to changes in MAIT cell numbers, the balance of MAIT cell functional subsets seems to also be relevant in the development of exacerbated forms of asthma, as MAIT17-like cells are enriched in the bronchoalveolar lavage (BAL) of children with severe asthma, and their frequency is associated with disease severity [73].

COPD is a major cause of morbidity worldwide, characterised by persistent airflow limitation accompanied by a chronic inflammatory immune response in the airways. As described in other lung diseases, frequency of circulating MAIT cells is reduced in COPD patients and correlates with the risk of hospitalisation [[74], [75], [76]]. This decrease is accompanied by an increase in MAIT cells in the lungs, suggesting a possible migration of cells from the circulation [76]. Moreover, in COPD MAIT cells (from blood and lung) show an activated phenotype and increased production of IL-17 accompanied by decreased IFN-γ (76). It is important to note however that another study suggested that the effect in MAIT cell numbers in COPD patients was dependent on administration of corticosteroids [77]. While in corticosteroid naïve COPD patients MAIT cell numbers remain unchanged, in corticosteroid-treated COPD patients MAIT cell frequencies are reduced in blood and endobronchial biopsy specimens. Moreover, *in vitro* experiments indicate that steroids have a direct effect in MAIT cell effector functions (reducing IFN-γ secretion), suggesting that corticosteroid treatment -rather than COPD itself- may influence MAIT cell numbers and function [77]. Thus, further studies are needed considering possible direct or indirect effects of treatments on MAIT cells in patients with COPD and other inflammatory diseases.

A limited number of studies have evaluated the possible roles of MAIT cells in CF. A key hallmark of CF is a persistent airway inflammation coupled with chronic bacterial infection driven by bacteria colonising the lung such as *Pseudomonas aeruginosa* which ultimately promotes an accelerated decline of lung function [78]. This pathogen has been shown to activate circulating human MAIT cells *in vitr*o [40] and a reduced frequency of MAIT cells has been reported in the peripheral blood of patients with CF, with reduced MAIT cell numbers correlating with *P. aeruginosa* infection as well as increased disease severity [79]. Moreover, a case report of a patient with CF characterised by severely impaired control of bacterial respiratory infections, showed a selective and near-complete deficiency on MAIT cells [80]. However, another report analysing unconventional T cells in the BAL of CF children showed no changes in the frequency of MAIT cells irrespective of age or infection status [81]. Thus, further studies are warranted to understand the circulating vs. pulmonary changes in MAIT cell populations in patients with CF and the relevance of these cells in controlling bacterial infections in the CF lung.

The role of MAIT cells in tumour immunity is only recently starting to emerge with reports suggesting both pro- and anti-tumour functions [82]. While data is scarce in the context of the lung, some studies suggest the participation of MAIT cells in lung cancer and metastasis. Contrasting results have been proposed regarding circulating MAIT cells in lung cancer patients with reports presenting an increase [83] or decrease [84] in MAIT cell frequency in lung cancer patients vs. healthy donors. Within the lung, MAIT cells have been shown to be enriched in the tumours of non-small cell lung cancer (NSCLC) patients (in relation to paratumour tissue) with high tumour infiltration of MAIT cells correlating with unfavourable disease-free survival [84]. Tumour infiltrating MAIT cells exhibited an exhausted phenotype with increased expression of exhaustion markers (PD1, TIM3) and decreased production of IFN-γ and Granzyme B. Moreover, circulating cytotoxic MAIT cells are increased in patients with NSCLC who respond to anti-PD-1 therapy, and show predictive value for response to immune-checkpoint inhibitors [84]. Preclinically, the *in vivo* functional role of MAIT cells in promoting or suppressing lung cancer progression has not been addressed, although several articles have suggested a contribution of MAIT cells in experimental mouse models of lung metastasis [51,85]. Intravenous injection of B16F10 melanoma cells resulted in reduced lung metastases in MR1-KO (vs. WT [85]) or B6-MAIT^CAST^MR1^-/-^ (vs. B6-MAIT^CAST^ [51]) mice indicating that MAIT cells promote lung metastasis. The effect of MAIT cells in this context is mediated by enhanced NK cell mediated anti-tumour immune responses suggesting a MAIT-NK cell crosstalk which may regulate tumour immunity.

## 7 Targeting pulmonary MAIT cells for therapy

Given the abundance and functional roles of MAIT cell in the airways, these cells are increasingly becoming attractive targets for the design of new therapeutic approaches. MAIT cell activation has been shown to augment adenovirus vector vaccine immunogenicity [86] which suggests the feasibility of targeting MAIT cells as cellular adjuvants for vaccine purposes. Indeed, intranasal administration of the MAIT cell antigen 5-OP-RU is sufficient to increase humoral responses against a co-administered protein antigen, providing a new approach for the induction of effective mucosal immunity [87]. This process is mediated by MAIT cell-dependent activation of dendritic cells, subsequent promotion of T follicular helper cell differentiation and induction of B cell responses [87]. Likewise, a specific population of T follicular helper-like MAIT cells has been shown to provide B cell help after mucosal challenge with *Vibrio cholerae* [88], further suggesting MAIT cells as potential targets for mucosal vaccines. MAIT cell antigens have also been used as adjuvants for vaccines against respiratory viruses, resulting in accumulation of MAIT cells in the lungs, increased vaccine efficacy and increased protection against viral pathogens [87,89]. Moreover, several studies in murine models have demonstrated that local expansion of pulmonary MAIT cells induced by the administration of 5-OP-RU together with TLR ligands or cytokines, is sufficient to afford protection from subsequent bacterial infection [32,33,39]. Likewise, 5-OP-RU mediated expansion of pulmonary MAIT cells provides protection against tumour lung metastases [51], suggesting that the MAIT cell population might be boosted for clinical benefit. While the use of MAIT cell antigens could provide a potential therapeutic approach, a recent study demonstrated that MAIT cells do not expand, and become functionally inactivated in response to 5-OP-RU administration in non-human primates [90], bringing into question whether a similar approach will be effective in humans. Alternatively, induced pluripotent stem-cell (iPSC)-derived MAIT cells have been recently generated [91] and human MAIT cells can be efficiently expanded *ex vivo* [92]. Since MAIT cells are not MHC-restricted (and devoid of alloreactive potential) they can potentially be used in universal adoptive therapy in a variety of contexts including refractory or hard-to-treat viral or bacterial infections or cancer. Also, these cells could be genetically manipulated to drive specific effector functions, homing to specific tissues or potentially kill infected or tumour cells [93]. These approaches emphasize the promising potential for advancing treatments by understanding and developing therapeutics based on MAIT cell manipulation.

## 8 Concluding remarks and open questions

Increasing evidence supports contributions of MAIT cells to the control of immunity in the airways in response to infections, during inflammatory lung diseases and in tumour immunity. Despite great advances, the future manipulation of MAIT cells for clinical applications requires further understanding of the mechanisms driving their activation and functional heterogeneity as well as the integration of murine and human data to obtain a complete picture of the MAIT cell immune response.

Amongst the many open questions in MAIT cell biology the identification of the mechanisms underpinning pulmonary MAIT cell activation and the effector functions by which they provide (or not) protection in disease remain key areas of investigation. This is particularly relevant in the context of inflammatory diseases and cancer where the signals driving MAIT cell functions remain obscure. MAIT cell activation can be mediated by MR1-dependent and independent mechanisms, although the contribution of each of these signals (e.g. TCR, cytokines, chemokines, co-stimulation) to activation and effector functions and how they influence the outcome of immunity still need to be determined. It is likely that such signals are uniquely linked to the type of insult (specific pathogen, inflammatory condition, tumour type) which will ultimately dictate the timeframe of the disease (acute vs chronic), the different activation/recruitment of immune cells and the cytokine milieu in the lung tissue. In conjunction to this, a variety of local signals (cytokines, chemokines) will also influence the specific location and migration of MAIT cells within the microanatomy of the lung shaping the speed and effectivity of their activation. On the other hand, the MAIT cell effector functions underpinning their protective (or detrimental) roles in disease remain also poorly defined, and whether distinct MAIT cell populations (e.g. MAIT1 vs MAIT17) will differently contribute to immune responses to distinct insults is an open question. The answer to these questions will be aided by murine models enabling targeted cell-specific deletion of specific molecules (MR1, costimulatory molecules, cytokines) or of distinct MAIT cell populations to ultimately determine the signals and cellular crosstalk controlling MAIT cell activation and functions in the various disease contexts. Finally, there is a pressing need for decoding the functions of human MAIT cells as well as the signals triggering their activation and effector functions in disease. While data for circulating MAIT cells is emerging, data for pulmonary cells is scarce, particularly in the diseased lung. Given the tissue-resident nature of MAIT cells the characterisation of the properties and effector functions of these cells within their tissue environment is of vital importance. Integration of human and murine data for pulmonary MAIT cells in various disorders will be critical to unequivocally define their protective or detrimental roles in disease and will support efforts to use this population in a therapeutic context.

## CRediT authorship contribution statement

**J.C. López-Rodríguez:** Writing – review & editing, Writing – original draft, Conceptualization. **P. Barral:** Writing – review & editing, Writing – original draft, Funding acquisition, Conceptualization.

## Declaration of competing interest

The authors declare the following financial interests/personal relationships which may be considered as potential competing interests: Patricia Barral reports financial support was provided by Biotechnology and Biological Sciences Research Council. If there are other authors, they declare that they have no known competing financial interests or personal relationships that could have appeared to influence the work reported in this paper.
